# Proxy- and Patient-Reported Outcome Measures in Cleft Lip and/or Palate Under 8 Years of Age: A Scoping Review

**DOI:** 10.1177/10556656251382058

**Published:** 2025-12-16

**Authors:** Kariym Joachim, Alexandra D’Souza, Jessie Howard, Aaron M. Drucker, Christopher R. Forrest, Karen W.Y. Wong Riff

**Affiliations:** 1 Institute of Medical Science, 12366 University of Toronto, Temerty Faculty of Medicine, University of Toronto, Toronto, Canada; 2 Division of Plastic and Reconstructive Surgery, 7979 The Hospital for Sick Children (SickKids), Toronto, Canada; 3 Department of Communication Disorders, The Hospital for Sick Children (SickKids), Toronto, Canada; 4 Division of Dermatology; Department of Medicine, 12366 University of Toronto, Toronto, Canada; 5 Institute of Health Policy, Management and Evaluation, 7938 University of Toronto, Toronto, Canada; 6 Department of Medicine and Research and Innovation Institute, 7985 Women's College Hospital (WCH), Toronto, Canada; 7 Institute for Clinical Evaluative Sciences (ICES), Toronto, Canada; 8 Division of Plastic, Reconstructive and Aesthetic Surgery, Department of Surgery, Temerty Faculty of Medicine, 7938 University of Toronto, Toronto, Canada; 9 Child Health and Evaluative Sciences, SickKids Research Institute, 7979 The Hospital for Sick Children (SickKids), Toronto, Canada

**Keywords:** outcomes, pediatrics, quality of life, systematic review, cleft lip, cleft palate

## Abstract

**Objective:**

This scoping review aimed to identify patient-reported outcome (PRO) and proxy-report outcome (ProxRO) measures administered to children under 8 years of age with cleft lip and/or palate (CL/P) and to describe the age at which proxy or self-report measures were used.

**Design:**

Scoping Review

**Setting:**

When children are unable to self-report, PROs and ProxROs can be used. The age at which proxy-report is appropriate has not been described in patients with CL/P.

**Patients, Participants:**

Children born with CL/P.

**Interventions:**

Ovid MEDLINE, EMBASE, PsycINFO, the Cochrane Register, and Web of Science were searched from inception until July 2024. Title and abstract screening, full text review, and data extraction were done independently in duplicate. Measures were categorized as PROs, ProxROs, or “MultiROs,” where both caregivers and children responded. A narrative synthesis was performed to describe the identified measures.

**Main Outcome Measures:**

PRO or ProxRO measures in children with CL/P less than 8 years old.

**Results:**

Of 7001 publications identified, 57 studies met the inclusion criteria. Fifty-six measures were identified, of which 43 had at least 1 published psychometric property. Thirteen studies used ad hoc measures. Most measures were not condition-specific to CL/P. ProxROs were more commonly utilized than PROs. Parent-proxy tools were used from birth to 8 years, while self-report was used in patients as young as 3 years old.

**Conclusions:**

There is a paucity of cleft-specific ProxRO measures that assess outcomes of cleft care in patients under 8 years of age. Work to develop and validate ProxRO measures for CL/P is needed.

## Introduction

Cleft lip and/or palate (CL/P) and its sequelae have been associated with various psychosocial issues, including disruptions in psychosocial functioning, decreased quality of life, self-esteem issues, and an increased risk of psychological and emotional issues.^1^

It is therefore important that healthcare providers (HCPs) and researchers assess the outcomes of treatment while identifying and responding to the unmet needs and preferences of people with CL/P and their families. One of the key methods to capture this information is through patient-reported outcome (PRO) and Caregiver Proxy-Reported Outcome (ProxRO) measures. These measures assess patient and caregiver perceptions of symptoms, functional status, and health-related quality of life (HRQoL) in a scientifically sound manner.^2,3^ The primary difference is that PRO measures are self-reported and directly ask the patient about their own health and well-being, while ProxRO measures ask another person (usually a caregiver) to report on the patient's health and well-being as if they were the patient. ProxRO measures are often used when patients are unable to self-report due to cognitive impairment or young age.^4^ Importantly, the proxy-report is distinct from the caregiver or observer-report in that the proxy-report asks the respondent to answer based on what they believe the patient thinks, whereas the observer-report asks what the observer thinks.

PRO/ProxRO measures provide HCPs and researchers with a better understanding of patient experience and satisfaction, thereby allowing them to improve the quality and effectiveness of care. Incorporation of these measures has led to benefits in the clinical management of patients, evaluation of care, and research, as well as improvements in communication and shared clinical decision-making.^2^ These outcome measures may be generic or condition-specific.^5^ The former is focused on health concepts that can be applied across many diseases or conditions, while the latter captures health outcomes unique to a particular disease or condition.^6^ Given the unique physiological and psychosocial impacts of CL/P, condition-specific PRO/ProxRO measures are necessary to capture the outcomes that matter most to people with CL/P, as well as to measure the impact of cleft-specific treatment.^5,7^

Systematic reviews of the literature to identify PROs previously used in children with CL/P have found few condition-specific PRO measures, which highlighted the need for validated PRO measures.^8,9^ In response to this gap, condition-specific PRO measures were developed,^10,11^ including the CLEFT-Q,^12^ which is designed for children and young adults with CL/P aged 8 to 29 years. The CLEFT-Q consists of 12 scales measuring satisfaction with appearance, facial function, and HRQoL. The CLEFT-Q's psychometric properties have been established, proving it to be reliable and valid in several studies to date.^12–15^ As the CLEFT-Q is only validated as a PRO measure in people with CL/P over 8 years of age, it cannot be used in younger children less than 8 years of age as is.^12^ The ability to measure concepts important to patients and families is therefore limited in this age group, thereby impeding the ability of HCPs to evaluate treatment outcomes. Furthermore, the reliability of self-report has been shown to be lower for children below 8 years of age,^16,17^ particularly, with respect to recall past 48 h and the limited ability of children in this age group to respond beyond dichotomous response options.^18^

Children with CL/P less than 8 years of age reach significant cognitive, language, emotional, and social development milestones^19^—all of which can be impacted by CL/P and its comorbidities. While they may be unable to self-report, children with CL/P in this age group have been reported to experience neurodevelopmental and academic deficits^20^ as well as difficulties with psychosocial adjustment.^1,21^ With respect to speech alone, by the age of 3 years, children with communication disorders have been reported to experience social isolation.^22^

There have not been many studies focusing on the perspectives of children with CL/P under age 8, with the majority of studies focusing on parents’ experiences.^23,24^ Given a significant amount of treatment of CL/P happens early in childhood, it is important to try to include the patient perspective as early as possible. Moreover, children under the age of 8 years have been described to have lower global self-concept and experience significant stigma.^25,26^ This would suggest that they are aware of the impact of having CL/P; thus, if they are undergoing treatment aimed at improving their HR-QOL, it is important to capture their perspective in the measurement of outcomes.

This study is a first step towards the multiphase adaptation of the CLEFT-Q into a caregiver ProxRO measure suitable for use in children with CL/P from birth to under 8 years of age. Development of a PRO involves (1) a literature review, (2) qualitative interviewing of the population of interest, and (3) stakeholder consultation.^27,28^ This literature review identifies available measurement tools and highlights gaps in measurement in order to assess the need for a new or revised measure for young children with CL/P.

The primary objective of this scoping review is to identify all PRO and ProxRO measures that have been used in children with CL/P under the age of 8 years. The secondary objective of this scoping review is to determine the age ranges for which PRO and ProxRO measures have been used.

## Methods

A preliminary search of MEDLINE, the Cochrane Database of Systematic Reviews, and JBI Evidence Synthesis was conducted, and no systematic reviews or scoping reviews on the topic were identified. This review is also a partial update to a 2012 systematic review, which identified PRO measures that assess quality of life in the CL/P literature.^8^ This scoping review was conducted in accordance with the Joanna Briggs Institute (JBI) methodology for scoping reviews^29^ and followed the Preferred Items for Systematic Reviews and Meta-analysis—Scoping Review (PRISMA-ScR)^30^ reporting guidelines.

### Eligibility Criteria

Studies were included if a PRO or ProxRO measure was shown to have been administered to children with CL/P less than 8 years of age or their primary caregivers. All forms of CL/P were included. The study had to indicate that at least a proportion of respondents were either the child themselves or their caregiver (eg, parent, legal guardian). If the sample did not include children under the age of 8 years, or age ranges were not stated, the study was excluded. No limits on context or study design were defined to capture a diversity of perspectives and experiences on the topic of interest.

Opinion papers and gray literature were not considered for inclusion as they do not provide complete information on the use of PRO or ProxRO measures. Systematic and scoping reviews were also excluded from this review. The reference lists of all excluded systematic and scoping reviews were examined, however, for eligible studies.

### Search Strategy

The search strategy was created to locate all published studies relating to our objectives with the assistance of a research librarian. The search strategy, including all identified keywords and index terms, was adapted for each included database and/or information source (Supplementary Table 1). Studies published in English were included. No date limits were applied. The initial search was completed on November 29, 2022. Databases included: Ovid MEDLINE, EMBASE, PsycINFO, the Cochrane Register and Web of Science. On July 17, 2024, the search was updated to ensure this scoping review would be current.

### Study/Source of Evidence Selection

Studies were uploaded and de-duplicated in Covidence, a web-based platform for systematic/scoping reviews and evidence synthesis.^31^ Three independent reviewers (A.D., J.H., and K.J.) screened the titles and abstracts against the review's inclusion/exclusion criteria. Two reviewers (A.D., J.H.) completed full-text screening at the following stage. Reasons for exclusion during full-text review were recorded. Any disagreement between the reviewers was resolved through discussion or with an additional reviewer (K.J.).

### Data Extraction

One reviewer (K.J.) extracted data from the included studies and iteratively updated a data-charting form through discussion with the study team. The data-charting form included the following study characteristics: study design, inclusion/exclusion criteria, sample characteristics (eg, age, types of CL/P), PRO or ProxRO measures used, and the constructs measured. The data-charting form can be found in Supplementary Table 2. Two independent reviewers (A.D. and J.H.) cross-checked and queried the extracted data. Any disagreements were resolved through discussion.

### Synthesis of Results

The measurement tools were grouped into 3 categories: (1) PRO, (2) ProxRO, and (3) MultiRO measures. If a measure was a self-report measure, it was categorized as a PRO measure. If a caregiver answered a measure as if they were their child, the measure was categorized as a ProxRO measure. Tools where parents respond as third-party observers of their child (rather than as if they were their child) are normally considered ObsRO measures. ObsRO measures ask caregivers to observe their child for outcomes of clinical importance.^4,32^ As not all studies provided copies of their questionnaires used, nor provided clear distinction between ProxRO and ObsRO measures, this study categorized all measures completed by parents under the category of ProxRO measures. Lastly, measures where caregivers and patients completed the same measure together or separately regardless of whether psychometric properties supported such use were categorized as “MultiRO” measures.

There are numerous measures (eg, PedsQL 4.0) that contain PRO and ProxRO versions of the same measure, or feature modules that assess separate constructs. As many of these measures will have differences in wording, domains, and items, they were categorized as different tools. For example, the PedsQL 4.0 Child Self-Report (Ages 5-7), the PedsQL 4.0 Parent Proxy-Report (Ages 5-7), and the PedsQL 4.0—Family Impact Module were counted as separate measures.^33,34^ PROs and ProxROs should have established psychometric properties in order to ensure scientifically sound measurement. As such, we recorded whether each measure had any published psychometric properties.^5^ Measures were categorized as having some supporting validation if there was evidence of at least 1 psychometric property; otherwise, the measure was categorized as having no supporting validation. In addition, the age range of participants completing each measure was identified.

## Results

**Figure 1.**
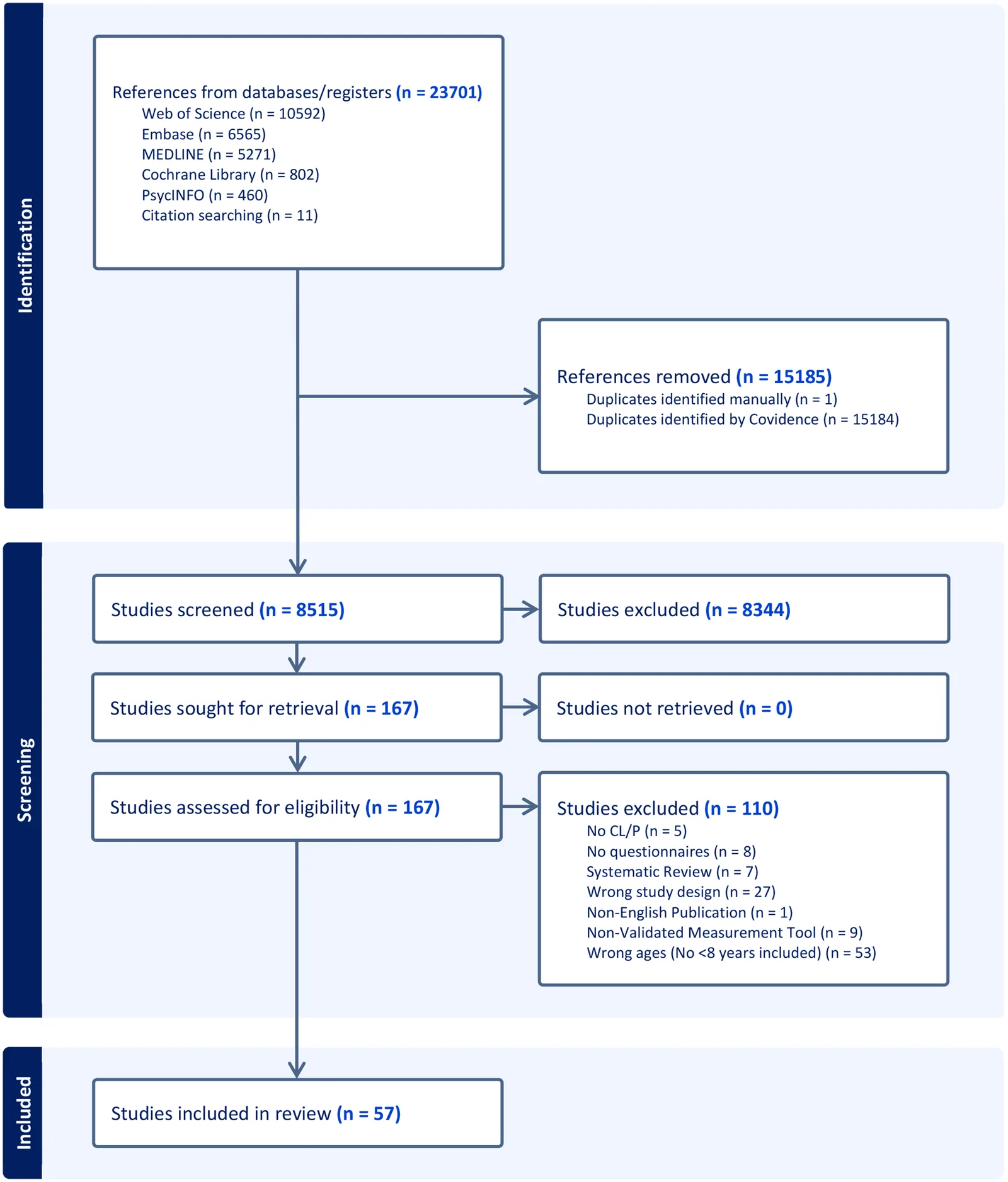
PRISMA flow diagram of included and excluded studies.

After de-duplication, 8515 studies were identified through the search strategy and the citation search of excluded systematic reviews (Figure 1). Title and abstract screening resulted in the removal of 8344 studies, leaving 167 studies for full-text assessment of eligibility. During full-text review, 110 studies were excluded. The remaining 57 studies met the inclusion criteria and were included in this scoping review.

The characteristics of each included study are described in Table 1, including country of origin, sample size, percentage of sample size living with CL/P, type of CL/P (ie, cleft lip and palate, cleft palate-only, cleft lip-only, cleft lip and alveolus), gender, age range of the sample, and study design.

**Table 1.** Characteristics of Included Studies.

Study ID	Country	Sample size	% with cleft	Type of cleft (%)	Gender (%)	Sample age range	Study design/type
Aarabi 2018**^35^**	Germany	396	8%	Unknown	M (47) F (53)	7 to 17 years	Cross-sectional
Ahmed 2024**^36^**	Pakistan	80	100%	CLP (60); CP (23); CL (18)	M (55) F (45)	6 to 19 years	Cross-sectional study
Al-Hassani 2024**^37^**	United Kingdom	659	100%	CLP (100)	M (68) F (32)	5 to 7 years	Cross-sectional study
Bhat 2019**^38^**	India	180	33%	CLP (100)	Unknown	0 to 30 years	Cross-sectional
Bickham 2017**^39^**	United States	168	100%	CLP (70); CP (30)	M (50) F (50)	5 to 19 years	Cross-sectional
Brand 2009**^40^**	Switzerland	66	48%	CLP (100)	M (70) F (30)	6 to 16 years	Case control
Branson 2024**^41^**	Australia	63	100%	CLP (46); CP (38); CL (10); CLA (6)	M (54) F (46)	4 to 17 years	Cohort study
Brichacek 2021**^42^**	Canada	37	100%	CL (100)	Unknown	0 to 6 years	Cross-sectional
Broder 1989**^25^**	United States	58	69%	CLP (33); CP (35); CL (33)	M (50) F (50)	7 years	Cross-sectional
Broder 2014**^43^**	United States	1200	100%	CLP (76); CP (24)	M (56) F (44)	7 to 19 years	Cohort
Broder 2017**^44^**	United States	1196	100%	CLP (76); CP (24); CL (0)	M (56) F (44)	7.5 to 18.5 years	Cohort
Bruneel 2019**^45^**	Belgium	30	83%	CLP (72); CP (28)	Unknown	3 to 10 years	Cross-sectional
Collett 2012**^46^**	United States	217	43%	CLP (36); CP (38); CL (26)	M (48) F (52)	5 to 9 years	Case control
Cook 2016**^47^**	United States	79	100%	CLP (90); CP (8); CL (3)	M (65) F (35)	4 to 10.9 years	Retrospective Chart Review
Costa 2021**^48^**	UK	322	100%	CLP (41); CP (38); CL (21)	M (42) F (58)	18 months	Cross-sectional
Damiano 2007**^49^**	United States	104	100%	CLP (48); CP (24); CL (28)	M (43) F (57)	2 to 12 years	Cross-sectional
DeJonge 2024**^50^**	United States	102	37%	CLP (68); CP (18); CL (8); CLA (5)	Unknown	1 to 16 years	Cross-sectional study
Edwards 2022**^11^**	United States	133	100%	CLP (74); CL (26)	M (66) F (34)	1.35 to 11.47 mo.	Development/Validation
Fadeybi 2012**^51^**	Nigeria	116	100%	CLP (24); CP (30); CL (46)	M (49) F (51)	0 to 64 years	Cross-sectional
Filgueira 2015**^52^**	Brazil	31	100%	CLP (39); CP (29); CL (32)	M (71) F (29)	2 to 5 years	Cross-sectional
Habibagahi 2024**^53^**	Iran	20	100%	Unknown	M (60) F(40)	42 mo. ± 4 mo.	Cohort study
Heike 2020**^54^**	United States	492	100%	CLP (37); CL (11)	Unknown	0 to 2 years	Development/Validation
Jokovic 2003**^55^**	Canada	231	NR	NR	M (45) F (55)	6 to 14 years	Development/Validation
Jones 2022**^56^**	United States	25	100%	CLP (72); CL (28)	M (64) F (36)	"Infant"	Cohort
Kapp-Simon 2024**^57^**	United States	123	100%	CLP (72); CLA (28)	M (34) F (66)	1.10 to 14.30 mo.	Cohort study
Khoun 2018**^58^**	Cambodia	133	32%	Unknown	M (47) F (53)	2 to 8 years	Cross-sectional
Kramer 2008**^59^**	Germany	147	100%	CLP (33); CP (31); CL (37)	M (59) F (41)	5 to 6.9 years	Cross-sectional
KrueckeBerg 1993**^60^**	United States	52	39%	CLP (25); CP (40); CL (35)	Unknown	3 to 6.75 years	Nonrandomized experimental study
Leopoldo-Rodado 2021**^61^**	Spain	357	48%	CLP (37); CP (30); CL (33)	M (51) F (49)	4 to 7.92 years	Case control
Lu 2022**^62^**	China	72	100%	CLP (100)	M (43) F (57)	4 to 20 years	Cross-sectional
Morgan 2020**^63^**	United States	75	59%	CLP (93); CL (7)	M (49) F (51)	7 to 21 years	Cross-sectional
Murray 2010**^64^**	UK	170	55%	CLP (67); CL (33)	M (62) F (38)	7 years	Cohort
Naros 2018**^65^**	Germany	134	100%	CLP (49); CP (32); CL (19)	M (52) F (48)	4 to 18 years	Cross-sectional
Nguyen 2019**^66^**	Vietnam, Estonia	56	100%	Unknown	Unknown	7 to 28 years	Cross-sectional survey
Nicholls 2018**^67^**	Australia	359	100%	CLP (47); CP (39); CL (14)	M (51) F (49)	6 to 42 years	Cross-sectional
Nicholls 2019**^68^**	Australia	359	100%	CLP (47); CP (39); CL (14)	M (51) F (49)	6 to 42 years	Cross-sectional
Pasini 2022**^69^**	Italy	64	50%	CLA (100)	M (66) F (34)	6 to 15 years	Cross-sectional
Patjanasoontorn 2012**^70^**	Thailand	36	100%	CLP (100)	M (50) F (50)	5 years	Cross-sectional
Prathanee 2024**^71^**	Thailand	15	100%	CLP (93); CL (7)	M (60) F(40)	3.83 to 10.75 years	Cohort study
Rahman 2024**^72^**	United States	131	100%	CLP (73); CL (27)	M (66) F (34)	"Infant"	Cohort study
Ramos 2022**^73^**	Brazil	106	100%	CLP (77); CP (12); CL (10)	M (59) F (41)	0 to 24 mo.	Nonrandomized experimental trial
Rando 2018**^74^**	Brazil	121	62%	CLP (100)	Unknown	2 to 6 years	Cross-sectional
Ranganathan 2016**^75^**	United States	93	100%	CLP (82); CL (18)	M (54) F (46)	5 to 20 years	Cross-sectional
Reissmann 2017**^76^**	Germany	140	16%	Unknown	M (51) F (49)	7 to 17 years	Cross-sectional
Rosenberg 2022**^77^**	United States	140	100%	CLP (73); CL (27)	M (4) F (96)	1.12 to 11.67 mo.	Cohort
Ruff 2017**^78^**	United States	327	25%	CLP (56); CP (26); CL (9)	M (46) F (54)	2 to 5 years	Cross-sectional
Sagheri 2009**^79^**	Germany	61	97%	CLP (47); CP (42); CL (10)	M (52) F (48)	4 to 7 years	Cross-sectional
Sander 2022**^80^**	Germany	152	100%	Unknown	M (59) F (41)	1 to 47 years	Development/Validation
Schonmeyr 2015**^81^**	India	134	100%	CLP (68); CP (32)	M (53) F (47)	7 to 35 years	Cross-sectional
Seifert 2021**^82^**	UK	412	100%	CLP (38); CP (36); CL (25)	M (64) F (36)	3 years	Cohort
Skirko 2015**^83^**	United States	57	56%	CLP (66); CP (34)	M (44) F (56)	3 to 22 years	Cohort
Soedjana 2022**^84^**	Indonesia	104	100%	CLP (74); CP (21); CLA (5)	M (57) F (43)	6 to 15 years	Cross-sectional
Thompson 2021**^85^**	New Zealand	378	100%	CLP (34); CP (43); CL (22)	M (46) F (54)	5 to 12 years	Cohort
Thompson 2021**^86^**	New Zealand	397	100%	CLP (35); CP (43); CL (22)	M (55) F (45)	5 to 12 years	Cohort
van Veen 2022**^87^**	Netherlands	58	100%	CLP (97); CP (3)	M (74) F (26)	0 to 8 years	Cross-sectional
Warschausky 2002**^88^**	United States	54	50%	CLP (74); CP (11); CL (7); CLA (7)	Unknown	5 to 18 years	Case control
Zeraatkar 2018**^89^**	Iran	110	50%	CLP (100)	Unknown	2 to 5 years	Cross-sectional

Studies included participants from 22 different countries, with the United States of America^11,25,39,43,44,46,47,49,50,54,56,57,60,63,72,75,77,78,83,88^ (*n* = 20), Germany^35,59,65,76,79,80^ (*n* = 6), Brazil^52,73,74^ (*n* = 3), and the United Kingdom^37,48,64,82^ (*n* = 4) represented most frequently. Sample sizes ranged from 15 to 1200 participants, and the age of participants with CL/P ranged from newborns up to 64 years.

### Characteristics of Measures

**Figure 2.**
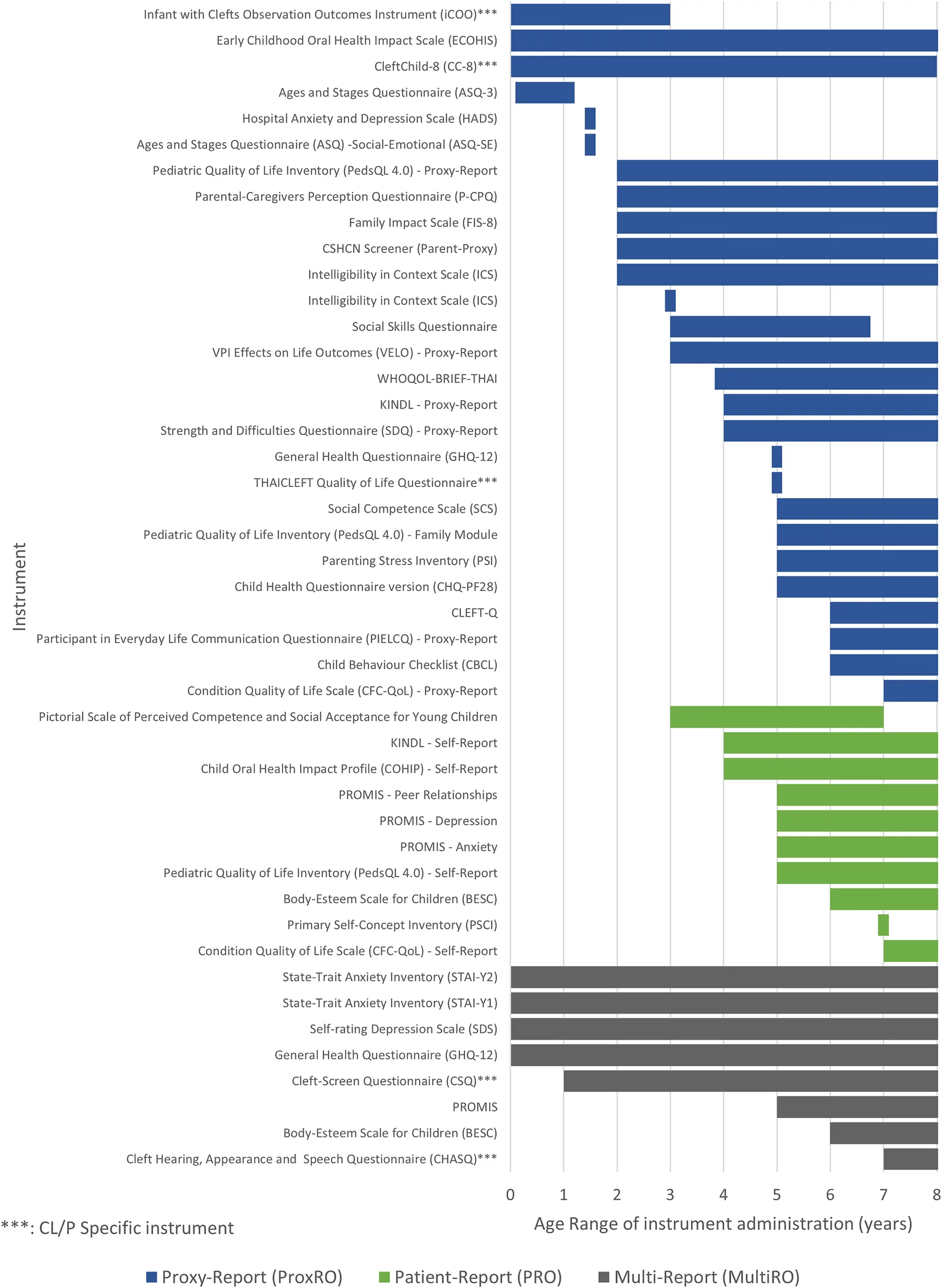
Age ranges where measures were administered as proxy-report (ProxRO), patient-report (PRO), or multi-report (MultiRO) for children with CL/P between birth and 9 years of age.

Fifty-six unique measures were identified. Of these, 13 were ad hoc measures that did not report any psychometric properties. The remaining 43 measures that reported at least 1 psychometric property are described in Table 2. Figure 2 displays the age ranges for which ProxRO, PRO, and MultiRO measures were given to children with CL/P and their families below the age of 8 years.

**Table 2.** Measures Used as Proxy-Report (ProxRO), Patient-Report (PRO), or Multi-Report (MultiRO) Outcomes.

		No. studies used as:		
Name of questionnaire	Constructs assessed	ProxRO	PRO	MultiRO
**Generic condition measures**				
Pediatric Quality of Life Inventory (PedsQL 4.0)—Proxy Report**^46,47,49,53,85,86^**	Health-related quality of life	6		
Early Childhood Oral Health Impact Scale (ECOHIS)**^50,52,73,74,89^**	Impact of oral health on child and family quality of life	5		
KINDL-p—Proxy Report**^59,61,65,79^**	Health-related quality of life	4		
Child Behaviour Checklist (CBCL)**^46,64,84^**	Emotional and behavioral adjustment	3		
Child Oral Health Impact Profile (COHIP)—Proxy Report**^44,76,78^**	Oral health-related quality of life	3		
Velopharyngeal Insufficiency (VPI) Effects on Life Outcomes (VELO)—Proxy Report**^45,62,83^**	Speech-related health-related quality of life	3		
Ages and Stages Questionnaire-3 (ASQ-3)**^48,57^**	Child development	2		
Parental-Caregivers Perception Questionnaire (P-CPQ)**^55,58^**	Impact of oral health on child and family quality of life	2		
Pediatric Quality of Life Inventory (PedsQL 4.0)—Family Impact Module**^85,86^**	Family functioning	2		
Strengths and Difficulties Questionnaire (SDQ)—Proxy Report**^40,41^**	Behavior, peer relations, emotional symptoms	2		
General Health Questionnaire (GHQ)**^51,70^**	Mental well-being	1		1
Ages and Stages Questionnaire (ASQ-SE)**^48^**	Child socio-emotional development	1		
Child Health Questionnaire version (CHQ-PF28)**^88^**	Health-related quality of life	1		
Children with Special Health Care Needs (CSHCN) Screener**^49^**	Special healthcare needs	1		
Condition Quality of Life Scale (CFC-QoL)—Proxy Report**^63^**	Health-related quality of life	1		
Family Impact Scale (FIS-8)**^58^**	Impact of oral health on child and family quality of life	1		
Hospital Anxiety and Depression Scale (HADS)**^48^**	Anxiety and depression	1		
Intelligibility in Context Scale (ICS)**^82^**	Speech intelligibility	1		
Parenting Stress Inventory (PSI)**^46^**	Parental functioning	1		
Participant in Everyday Life Communication Questionnaire (PIELCQ)—Proxy Report**^40^**	Communicative competencies	1		
Social Competence Scale (SCS)**^46^**	Social competence	1		
Social Skills Questionnaire**^60^**	Social skills	1		
WHOQOL-BRIEF-THAI**^71^**	Health-related quality of life	1		
Child Oral Health Impact Profile (COHIP)—Self-Report**^35,43,44,61,69,76^**	Oral health-related quality of life		6	
Pediatric Quality of Life Inventory (PedsQL 4.0)—Self-Report**^46,47,75,85,86^**	Health-related quality of life		5	
KINDL—Self-Report**^59,61,65^**	Health-related quality of life		3	
Pictorial Scale of Perceived Competence and Social Acceptance for Young Children**^60,64^**	Self-concept/self-esteem		1	
Body-Esteem Scale for Children (BESC)**^67,68^**	Self-concept/self-esteem		1	1
Condition Quality of Life Scale (CFC-QoL)—Self-Report**^63^**	Health-related quality of life		1	
Primary Self-Concept Inventory (PSCI)**^25^**	Self-concept/self-esteem		1	
PROMIS—Anxiety**^75^**	Psychosocial functioning		1	
PROMIS—Depression**^75^**	Depression symptoms		1	
PROMIS—Peer Relationships**^75^**	Social functioning		1	
PROMIS**^39^**	Health-related quality of life			1
Self-rating Depression Scale (SDS)**^51^**	Depression			1
State-Trait Anxiety Inventory (STAI-Y1)**^51^**	Immediate anxiety			1
State-Trait Anxiety Inventory (STAI-Y2)**^51^**	General anxiety			1
**CLP-specific measures**				
Infant with Clefts Observation Outcomes Instrument (iCOO)**^11,54,56,72,77^**	Infant/child cleft-related health and well-being	5		
CleftChild-8 (CC-8)**^87^**	Quality of life, patient satisfaction	1		
THAICLEFT Quality of Life Questionnaire**^70^**	Quality of life	1		
CLEFT-Q**^36^**	Health-related quality of life (2 subscales)		1	
Cleft Hearing, Appearance and Speech Questionnaire (CHASQ)**^66^**	Satisfaction with hearing, appearance, and speech			1
Cleft-Screen Questionnaire (CSQ)**^80^**	Health-related quality of life			1
		**52**	**23**	**8**

Of the 43 measures identified in this scoping review, the majority (*n* = 37) were generic rather than CL/P-specific measures. There were 6 measurement tools created specifically for children with CL/P : (1) Cleft Hearing, Appearance and Speech Questionnaire (CHASQ),^66^ (2) CleftChild-8 (CC-8),^87^ (3) Cleft-Screen Questionnaire (CSQ),^80^ (4) Infant with Clefts Observation Outcomes Instrument (iCOO),^11,54,56,77^ (5) THAICLEFT Quality of Life Questionnaire,^70^ and the (6) CLEFT-Q.^36^ One measure (the Velopharyngeal Insufficiency Effects on Life Outcomes or VELO) was specific to velopharyngeal insufficiency, which can be related to CL/P but is not necessarily associated.^90^ Of all the cleft-specific measures, only the THAICLEFT study was published more than 5 years preceding this scoping review.

#### ProxRO Measures

Twenty-six measures were ProxRO measures. The most often used ProxRO measure was the Pediatric Quality of Life Inventory (PedsQL 4.0) parent-proxy measure in 6 studies,^46,47,49,53,85,86^ followed by the Early Childhood Oral Health Impact Scale (ECOHIS),^50,52,73,74,89^ and Infant with Clefts Observation Outcomes Instrument (iCOO)^11,54,56,72,77^—each used in 5 studies. Most of these measures (12/26 measures) were used to assess quality of life (QoL) or health-related quality of life (HRQoL).

ProxRO measure usage ranged from birth to 8 years of age. Three of the aforementioned 6 cleft-specific measures were used as ProxRO measures—the CleftChild-8 (CC-8),^87^ (newborn to 8 years old), Infants with Clefts Observation Outcomes Instrument (iCOO)^11,54,56,77^ (newborn to 3 years old), and the THAICLEFT Quality of Life Questionnaire^70^ (5 years of age only). The average lower age limit for ProxROs was 3.2 years.

#### PRO Measures

Eleven measures were PRO measures. Among these tools, the Child Oral Health Impact Profile (COHIP) was administered most (6 studies),^35,43,44,61,69,76^ followed by the PedsQL 4.0 child self-report measure (5 studies),^46,47,75,85,86^ the Kiddy/Kid/Kiddo KINDL (3 studies).^59,61,65^ Again, QoL/HRQoL was the most assessed construct (5/11 measures), though self-concept/self-esteem (3/11 measures) was often investigated. PRO measures were used in patients as young as 3 years old through age 8 years. This review identified only 1 cleft-specific measure used as a PRO for children under the age of 8 years—the CLEFT-Q.^36^ The average lower age limit for PRO measures (5.2 years) was higher than that of ProxRO measures.

#### MultiRO Measures

Eight of the included tools were used at least once as a MultiRO. Two measures (the General Health Questionnaire and the Body-Esteem Scale for Children) were used in 2 studies respectively as PRO measures and again in 2 separate studies as MultiRO measures. Mental health (3/8 measures), QoL/HRQoL (2/8 measures), and self-concept/self-esteem (2/8 measures) were the 2 concepts most assessed by MultiROs.

#### Measure Summary

Of particular interest to this scoping review are the measures that were at least partially validated for use in children with CL/P. The ages of the patients in which these measures were used is described.

##### CleftChild-8 (CC-8)

The CleftChild-8 (CC-8) ProxRO measure assesses domains of CL/P-specific health-related quality of life and patient satisfaction in children.^91^ The 43-item questionnaire assesses 4 domains on 4 subscales: (1) parental satisfaction with the functioning of the cleft team, (2) satisfaction with surgery, (3) social functioning, and (4) psychological functioning. This review identified 1 paper where the CleftChild-8 was administered as a ProxRO measure in children ranging from newborn to 8 years of age.^87^

##### Cleft Hearing, Appearance, and Speech Questionnaire (CHASQ)

The Cleft Hearing, Appearance, and Speech Questionnaire (CHASQ) is a 15-item PRO measure that assesses patients’ satisfaction with features of appearance, speech, and hearing using 2 domains.^92^ The first domain captures patient perspectives on cleft-associated features (eg, whole appearance, lips, hearing), while the second domain is comprised of items that assess other facial features (eg, hair, eyes, cheeks).^92^ The CHASQ was administered in 1 identified paper to patients aged 7 to 28 years and/or their primary caregiver.^66^

##### Infants With Cleft Observation Outcomes Instrument (iCOO)

The Infants with Cleft Observation Outcomes Instrument (iCOO) is an ObsRO measure,^11^ rather than a ProxRO measure, meaning that it is designed to assess the observer's opinion of the outcome of interest. Consisting of 65 items, the iCOO assesses 9 domains: (1) behavioral and emotional health, (2) breathing, (3) comfort, (4) communication, (5) ears and hearing, (6) facial skin and mouth, (7) feeding, (8) sleep, and (9) vocalization. This review identified 5 papers where the iCOO was deployed as a ProxRO measure for infants from newborn to 3 years of age.^11,54,56,72,77^

##### Cleft Screen Questionnaire (CSQ)

The Cleft Screen Questionnaire (CSQ) is a 38-item screening measure that was created to be a succint, yet comprehensive cleft-specific screening measure for HRQoL in individuals with CL/P. Consisting of 38 items, the CSQ's items group into 5 different domains of which have not yet been interpreted.^80^ One paper identified in this review administered the CSQ to patients between 1 and 47 years of age as a PRO or ProxRO dependent on patient age.^80^

##### Thai Cleft Quality of Life Questionnaire (THAICLEFT QOL)

The Thai Cleft Quality of Life Questionnaire (THAICLEFT QOL) is intended to be a ProxRO measure that captures the quality of life of children with CL/P living in a Thai sociocultural context. The number of scale items in the THAICLEFT questionnaire is unclear, with 2 studies stating that the total number of items is 20^70^ or 24.^93^ The THAICLEFT QoL was developed by extracting and modifying items from a number of generic measures (eg, KINDL, WHOQOL-BREF, and Impact on Family Scale) and notes from the 2009 Thai Cleft Lip Palate and Craniofacial Association.^93^ The THAICLEFT measure was administered as a ProxRO in 1 paper identified in this review to primary caregivers of children 5 years of age.

##### CLEFT-Q

The CLEFT-Q is a condition-specific PRO measure for use in children with CL/P between 8 to 29 years of age.^12,15,94,95^ This review identified 1 paper that reported using 2 CLEFT-Q subscales—Psychological Function and Social Function—among children ages 6 to 19 years.^36^

## Discussion

In this scoping review, we identified 57 primary studies where PRO and ProxRO measures were utilized among children under 8 years of age living with CL/P. We identified a total of 43 different tools that had undergone some level of psychometric testing and validation. The findings altogether suggest a widespread interest in capturing outcomes beyond conventional biomarkers and clinician-reported outcomes (ClinROs) measures in studies of CL/P.

Utilizing the HCP perspective and expertise to assess a patient's health status (eg, rating of speech intelligibility, pre- and postsurgery anthropometrics) through ClinRO measurements is a cornerstone of medicine.^32^ ClinRO measures, however, cannot assess patient and family perspectives on the physical and psychosocial health of a child during CL/P treatment.^95^ This review highlights PRO and ProxRO measures as valuable tools to capture patient and family perspectives, as these perspectives are often undervalued in favor of ObsRO and ClinRO measures.^96–98^ PRO and ProxRO measures directly assess how patients and families feel and function by eliciting patient or caregiver perspectives. In contrast, ClinRO measures and biomarkers assess the same outcomes but through the perspective and interpretation of HCPs who see their patients episodically rather than throughout their daily lives.^99^ PRO and ProxRO measures also capture what is important to patients and families while avoiding observer bias, while ClinRO measures often assess outcomes that are important to HCPs.^99^ Consequently, PRO and ProxRO measures can help HCPs assess patient needs, improve care quality, track progress and outcomes, and adapt interventions accordingly. These outcome measures also foster communication and shared decision-making among patients, families, and HCPs.^2^

Though there is a widespread interest in non-ClinRO measures with respect to CL/P, the majority of the measures included in this review were not condition-specific. As a result, many of the biophysiological, clinical, and psychosocial sequelae related to CL/P may not be captured in a clinically meaningful, scientifically sound manner. Many generic measures do not include content that is relevant to patients with CL/P, and if these measures are used to assess outcomes, changes in concepts that are not included will not be assessed.

The closest generic measures to capturing CL/P related outcomes are the ones that queried HRQoL—an outcome that is often impacted heavily by a CL/P diagnosis. The most pertinent measures used as ProxRO measures would be the Child Oral Health Impact Profile (COHIP), the Early Childhood Oral Health Impact Scale (ECOHIS), the Parental-Caregivers Perception Questionnaire (P-CPQ), and the Family Impact Scale (FIS-8). These measures can be used to capture the impact of a child's oral condition on the child, their family, or both. However, they are still limited in that their focus is primarily on the impacts of gum and tooth health and less on the impacts of CL/P on the domains of speech, eating, drinking, biophysiological development, appearance, and psychosocial growth.

We also found that there were relatively few measures that were used as PRO measures. Of the 11 PRO measures, the majority limited self-report to children aged 5 years and up. Varni et al.^16^ have reported that the ideal age for self-report is 8 years and older.

One limitation among the 57 identified studies was that measures were administered to age groups for which they were not validated for use. For example, a 14-item Spanish-translated version of the COHIP (COHIP-14) was deployed as a PRO measure in patients aged 4 to 7 years of age,^61^ although the COHIP-14 was validated in children age 12 to 21 years of age.^100^ The CLEFT-Q was also administered as a PRO measure in patients aged 6 to 19 years of age,^36^ when the CLEFT-Q has been validated in individuals 8 to 29 years of age.^12^

Another inherent issue is with MultiRO measures—self-report PRO measures that were administered to caregivers without modification, and where caregivers were asked to either complete on their own behalf,^51^ on their child's behalf,^80^ or together with their child.^67,68^ In these situations, cross-informant variance between self-report and proxy-report and bias threaten measure validity and interpretability.^17,80^ To avoid this, patients and caregivers should be given separate but similar validated measures that assess the same constructs with parallel items.^17^

Below, we briefly summarize each of the 6 identified measures below in relation to the conceptual framework of Wong et al.^95^ Developed through interviews with individuals living with CL/P, this conceptual framework reflects the issues that individuals with CL/P and their families (rather than HCPs) determined to be most important to measure.^95^ These issues regard CL/P outcomes and are divided into 3 domains: (1) appearance outcomes (ie, face, lips, nose, nostrils, cleft scar, teeth, jaws), (2) health-related quality of life (ie, speech-related distress, psychological function, social function, school function), and (3) facial function (ie, speech function, eating, and drinking).^95^

### CleftChild-8 (CC-8)

Formerly known as the K(N)UZS-8,^91^ strengths of the CleftChild-8 (CC-8) include the engagement of parents and support groups during its development, as well as suitable internal consistency, test-retest reliability, and construct validity.^87,91^ The domains of the CC-8, however, focus more on process outcomes. Half of the domains and subdomains are focused on satisfaction with the cleft team or cleft care, while key outcomes identified by Wong et al.^95^ such as satisfaction with appearance, speech-related distress, or facial function, are not included. Moreover, the CC-8 has only been used once in a publication since its development in 2016,^87^ and has not yet been translated from Dutch into other languages.

### Cleft Hearing, Appearance, and Speech Questionnaire (CHASQ)

A full validation study on the CHASQ has yet to be published,^101^ though psychometric testing of successive CHASQ translations suggests that it shows satisfactory internal validity, construct validity, and overall psychometric properties in patients over the age of 7 years.^10,66,92,101,102^ There is, however, a lack of consistency in how the CHASQ is administered or interpreted—some studies have used a single-item version of the CHASQ while other studies have used the full 15 items.^103^ There is also uncertainty in whether the CHASQ is solely a PRO measure as described by Stiernman et al.^92^ whether there is a ProxRO version, or whether caregivers are given the PRO measure and asked to answer on behalf of their child.^66^ For this reason, we categorized the CHASQ as a MultiRO measure.

The “satisfaction with cleft-related features” domain aligns with some aspects of the “Appearance” domain that patients with CL/P identified as important to measure.^95^ The CHASQ does not assess health-related quality of life, emotional-well-being, or other impacts of CL/P on their lives. In a comparative study between the CLEFT-Q and the CL/P, respondents found it easier and faster to complete the 1-scale CHASQ compared to the 12-scale CLEFT-Q, but still preferred the depth and detail of the CLEFT-Q scales and thought they would provide better detailed and broader information to HCPs.^104^ Despite the relative ease and speed of completing the CHASQ, additional work needs to be done to clarify its format (ie, PRO vs ProxRO) as well as its psychometric properties as a ProxRO for children with CL/P under 8 years.

### Infants With Cleft Observation Outcomes Instrument (iCOO)

The psychometric properties of the Infants with Cleft Observation outcomes Instrument (iCOO) have been established for use in newborn children with CL/P up until 3 years of age and have shown good internal consistency, construct validity, structural validity, and responsiveness.^11^ The goal of the iCOO is to reliably record caregiver observations of CL/P-related clinical signs so that HCPs can better assess child health.^54^ The iCOO was developed using robust methodology with caregiver input and feedback.^54^ It is a useful tool for the age group for which it is designed, when patients themselves are unlikely to be able to self-report. The caregiver perspective is important to measure and understand in tandem with the patient perspective. Above the age of 3 years, patients may start to understand some of the concepts of interest to health care providers. For the measurement of this patient perspective at a young age (for example, being able to be understood at school or pre-school), a proxy-report measure would be ideal.

### Cleft Screen Questionnaire (CSQ)

Some of the CSQ's psychometric properties have been published, showing good internal consistency in a sample of people with CL/P from 1 year to 47 years old and their parents.^80^ Borumand et al.^105^ highlight that the CSQ's development process is incomplete, as only a principal components analysis (PCA) was performed when an exploratory factor analysis should have been undertaken. Moreover, the CSQ's developers opted not to completely establish the measure's psychometric properties, as it was presumed that the CSQ's items (which had been adapted from 14 already-existing questionnaires) were already valid.^80,105^ The CSQ is designed to be a self-report PRO measure, but parents were asked to respond on their child's behalf when they were too young. Of note, “young” was not defined as a specific age group in this case. As with the other MultiROs identified in this scoping review, cross-informant variance is an issue with ramifications for bias, validity, and interpretability. Without complete reporting of the measure's psychometric properties, however, it is difficult to know if the CSQ is valid as either a PRO or ProxRO measure.

### Thai Cleft Quality of Life Questionnaire (THAICLEFT QOL)

The THAICLEFT QOL—similar to the CSQ—has had some psychometric testing, showing good internal consistency in a sample of parents of children with CL/P.^70,93^ There was no information published on the ages of the children in the sample. The THAICLEFT QOL did not involve patients or families in the development process. The majority of questions contained in the THAICLEFT QOL focus on the quality of life, mental health, and needs of the parents as opposed to the child,^70^ making this neither a PRO nor a ProxRO measure, but a more general measure of caregiver and family health. Altogether, the lack of reporting of both the measure's development and its intended use, as well as its focus on caregiver outcomes, makes this measure less useful as a ProxRO measure in children with CL/P under 8 years.

### CLEFT-Q

Though its psychometric properties have been established repeatedly in individuals 8 to 29 years of age, the CLEFT-Q is not recommended for use below 8 years of age as younger children are unable to reliably self-report HRQoL.^16,17^ The usage of the CLEFT-Q below its validated age range further suggests that there is a need for a validated version of the CLEFT-Q for children under 8 years of age.

### Age of PRO and ProxRO Usage

Our review found that PROs were administered to children with CL/P as young as 3 years of age, despite research suggesting that the results of PROs are unreliable at this age.^16^ Conversely, a ProxRO version of a measure was administered alongside a PRO version in patients as old as 22 years.^83^ This implies a lack of clarity as to what age a child should be completing a PRO as opposed to their primary caregiver completing a ProxRO. Guidelines are needed regarding why and when it is appropriate to use a ProxRO or a PRO, and at what age that children should be approached for self-report.^4,106^ ProxROs should only be used when a child is otherwise unable to complete a PRO^17^—using a ProxRO when a child is able to self-report has implications for the reliability and validity of longitudinal research.

### Limitations

Our team acknowledges the limitations of our scoping review. Our review was limited by the paucity of condition-specific PRO and ProxRO measures available for our target population. In addition, heterogeneity in the reporting of measure development and the establishment of psychometric properties was a limitation in interpreting the results of our review. Finally, as scoping reviews are exploratory and intended to capture the broader scope of the literature, critical appraisal of the study methodology and quality were not part of our review.

## Conclusions

This scoping review identified 6 cleft-specific measures that could potentially be used to capture outcomes of children with CL/P under the age of 8 years. Of these 6 tools, the CleftChild-8 (CC-8) is the only ProxRO measure that covers the full age range of interest. Its focus on process-related outcomes, however, means that the CC-8 may not assess the outcomes that matter most to individuals with CL/P and their families. As a result, there are no ProxRO measures that capture concerns that matter most to children with CL/P below the age of 8 years.

To fill this gap, either a new tool must be developed or a preexisting tool must be adapted. If adaptation of an existing tool is the way forward, the process should follow guidelines similar to the development of the initial tool itself. This may include in-depth qualitative interviews with parents and HCPs, adaptation of items for proxy-report, and large-scale field-testing. The final product should be a tool that HCPs and researchers can administer to caregivers of children 3 to 8 years of age with CL/P and capture health-related quality of life, satisfaction with appearance, and facial function, as defined by the conceptual framework of Wong et al.^95^ As per Varni et al.^17^ the adapted measure would need to mirror the existing tool, allowing for life-long, uninterrupted longitudinal assessment from birth through to adulthood.

## Supplemental Material

sj-docx-1-cpc-10.1177_10556656251382058 - Supplemental material for Proxy- and Patient-Reported Outcome Measures in Cleft Lip and/or Palate Under 8 Years of Age: A Scoping Review Supplemental material, sj-docx-1-cpc-10.1177_10556656251382058 for Proxy- and Patient-Reported Outcome Measures in Cleft Lip and/or Palate Under 8 Years of Age: A Scoping Review by Kariym Joachim, Alexandra D’Souza, Jessie Howard, Aaron M. Drucker, Christopher R. Forrest and Karen W.Y. Wong Riff in The Cleft Palate Craniofacial Journal

sj-docx-2-cpc-10.1177_10556656251382058 - Supplemental material for Proxy- and Patient-Reported Outcome Measures in Cleft Lip and/or Palate Under 8 Years of Age: A Scoping Review Supplemental material, sj-docx-2-cpc-10.1177_10556656251382058 for Proxy- and Patient-Reported Outcome Measures in Cleft Lip and/or Palate Under 8 Years of Age: A Scoping Review by Kariym Joachim, Alexandra D’Souza, Jessie Howard, Aaron M. Drucker, Christopher R. Forrest and Karen W.Y. Wong Riff in The Cleft Palate Craniofacial Journal
