# Supplementary material for: Proxy- and Patient-Reported Outcome Measures in Cleft Lip and/or Palate Under 8 Years of Age: A Scoping Review
Source: Cleft Palate Craniofac J. 2025 Dec 16;63(10):3176–91. doi: 10.1177/10556656251382058 (PMC13542404; doi:10.1177/10556656251382058)
Supplement: Supplementary file 1 [file sj-docx-1-cpc-10.1177_10556656251382058.docx]

### Supplementary Table 1: Search strategy (November 29, 2022)

**Literature Search Strategy**

| **Date** | November 14, 2022 |
| --- | --- |
| **Requester(s)** | -----, ----- |
| **Topic** | In patients with cleft lip and/or palate, less than 8 years of age, what patient-reported, caregiver proxy-reported, or parent-reported outcome measures have been used to evaluate outcomes of cleft care, and at what age was each type of outcome measure used? |
| **Date limit(s)** | n/a |
| **Language** | English |
| **Other limits** | Human; Omit conference abstracts and other less relevant publication types (embase, cinahl, web of science) |

| **Database [Platform]** *Searches run November 29, 2022* | **Results** |
| --- | --- |
| Ovid MEDLINE(R) and Epub Ahead of Print, In-Process, In-Data-Review & Other Non-Indexed Citations and Daily <1946 to November 28, 2022> | 2488 |
| Embase Classic+Embase [OVID] 1947 to 2022 week 47 | 3040 |
| PsycINFO – 1806 to November week 3 2022 | 217 |
| Cochrane Central Register of Controlled Trials – October 2022 | 359 |
| Web of Science(to be translated) | 4954 |
| **TOTAL* not deduplicated** | **11,058** |

(22118856 or 22030513) - Klassen et al, Eckstein et al

Search strategy:

Set 1 and Set 2

***Medline***

Set 1 Cleft palate management

Exp Cleft Lip/ or exp Cleft Palate/

or

((patient care or primary care or family medicine or family physician* or family doctor* or GP or general pract* or p$ediatr* or nurse or nursing or dental* or Speech or language or hearing or voice or vocal or dental or nutrition* or diet* or surgery or surgical or interdisciplinary or multidisciplinary or care* or manag*) and (cleft palate* or cleft lip$ or CLP or "CL/P" or "cl/cp")).tw,kw,kf

or

(palatoplast* or palatal repair* or Cleft Palate repair* or cleft lip repair* or cleft care*).tw,kw,kf

Set 2 Caregiver-, patient-, or proxy-reported outcomes

*"Patient Reported Outcome Measures"/ or *"patient satisfaction"/ or *"Surveys and Questionnaires"/ or *benchmarking/ or *" treatment outcome"/ or *"Outcome Assessment, Health Care"/ or ("patient-reported outcome measure*" or "PROM$" or PROMIS or survey$ or questionnaire* or instrument or measurement or "treatment outcome*" or "healthcare outcome*" or "health care outcome*").tw,kw,kf

Or

*proxy/ or *caregivers/ or ((proxy or proxies or caregiver or carer* or parent$) and (reported outcome*)).tw,kw,kf

or

("CleftQ" or "Cleft-Q").mp

or

*"Quality of Life"/ or (((quality adj2 life) or QoL or craniofacial* or voice* or "oral health") and (measure* or instrument)).tw,kw,kf

***Embase***

Set 1 Cleft palate management

Exp Cleft Lip/ or exp Cleft Palate/

or

((patient care or primary care or family medicine or family physician* or family doctor* or GP or general pract* or p$ediatr* or nurse or nursing or dental* or Speech or language or hearing or voice or vocal or dental or nutrition* or diet* or surgery or surgical or interdisciplinary or multidisciplinary or care* or manag*) and (cleft palate* or cleft lip$ or CLP or "CL/P" or "cl/cp")).tw,kw,kf

or

(palatoplast* or palatal repair* or Cleft Palate repair* or cleft lip repair* or cleft care*).tw,kw,kf

Set 2 Caregiver-, patient-, or proxy-reported outcomes

*"Patient-Reported Outcome"/ or *"patient satisfaction"/ or *"Questionnaire"/ or *benchmarking/ or *" treatment outcome"/ or *"Outcome Assessment"/ or ("patient-reported outcome measure*" or "PROM$" or PROMIS or survey$ or questionnaire* or instrument or measurement or "treatment outcome*" or "healthcare outcome*" or "health care outcome*").tw,kw,kf

Or

*proxy/ or *caregiver/ or ((proxy or proxies or caregiver or carer* or parent$) and (reported outcome*)).tw,kw,kf

or

("CleftQ" or "Cleft-Q").mp

or

*"Quality of Life"/ or (((quality adj2 life) or QoL or craniofacial* or voice* or "oral health") and (measure* or instrument)).tw,kw,kf

***PsycINFO***

Set 1 Cleft palate management

exp Cleft Palate/

or

((patient care or primary care or family medicine or family physician* or family doctor* or GP or general pract* or p$ediatr* or nurse or nursing or dental* or Speech or language or hearing or voice or vocal or dental or nutrition* or diet* or surgery or surgical or interdisciplinary or multidisciplinary or care* or manag*) and (cleft palate* or cleft lip$ or CLP or "CL/P" or "cl/cp")).tw,id,mh

or

(palatoplast* or palatal repair* or Cleft Palate repair* or cleft lip repair* or cleft care*).mp

Set 2 Caregiver-, patient-, or proxy-reported outcomes

*"Patient-Reported Outcome Measures"/ or *"client satisfaction"/ or *"Questionnaires"/ or *Best Practices/ or *"treatment outcomes"/ or *"health outcomes"/ or ("patient-reported outcome measure*" or "PROM$" or PROMIS or survey$ or questionnaire* or instrument or measurement or "treatment outcome*" or "healthcare outcome*" or "health care outcome*").tw,id,mh

Or

parents/ or caregivers/ or ((proxy or proxies or caregiver or carer* or parent$) and (reported outcome*)).tw,id,mh

or

("CleftQ" or "Cleft-Q").mp

or

*"Quality of Life"/ or (((quality adj2 life) or QoL or craniofacial* or voice* or "oral health") and (measure* or instrument)).tw,id,mh

***Cochrane Central Register of Controlled Trials***

Set 1 Cleft palate management

Exp Cleft Lip/ or exp Cleft Palate/

or

((patient care or primary care or family medicine or family physician* or family doctor* or GP or general pract* or p$ediatr* or nurse or nursing or dental* or Speech or language or hearing or voice or vocal or dental or nutrition* or diet* or surgery or surgical or interdisciplinary or multidisciplinary or care* or manag*) and (cleft palate* or cleft lip$ or CLP or "CL/P" or "cl/cp")).tw,kw,kf

or

(palatoplast* or palatal repair* or Cleft Palate repair* or cleft lip repair* or cleft care*).tw,kw,kf

Set 2 Caregiver-, patient-, or proxy-reported outcomes

Exp "Patient Reported Outcome Measures"/ or exp "patient satisfaction"/ or exp "Surveys and Questionnaires"/ or exp benchmarking/ or exp " treatment outcome"/ or exp "Outcome Assessment, Health Care"/ or ("patient-reported outcome measure*" or "PROM$" or PROMIS or survey$ or questionnaire* or instrument or measurement or "treatment outcome*" or "healthcare outcome*" or "health care outcome*").tw,kw,kf

Or

Exp proxy/ or exp caregivers/ or ((proxy or proxies or caregiver or carer* or parent$) and (reported outcome*)).tw,kw,kf

or

("CleftQ" or "Cleft-Q").mp

or

exp "Quality of Life"/ or (((quality adj2 life) or QoL or craniofacial* or voice* or "oral health") and (measure* or instrument)).tw,kw,kf

***Web of Science***

Set 1 Cleft palate management

TS=(( "cleft palate*" or "cleft lip$" or palatoplast* or palatal repair* or Cleft Palate repair* or cleft lip repair* or cleft care*))

Set 2 Caregiver-, patient-, or proxy-reported outcomes

TS=(("patient-reported outcome measure*" or "PROM$" or PROMIS or survey$ or questionnaire* or instrument or measurement or "treatment outcome*" or "healthcare outcome*" or "health care outcome*")

Or

TS=((proxy or proxies or caregiver or carer* or parent$ AND reported outcome*))

or

TS=(("CleftQ" or "Cleft-Q"))

or

TS=(("Quality of life" or QoL or craniofacial* or voice* or "oral health" AND measure* or instrument))

NOT

TS=((Animal OR animals or Animal Experiment* OR animal model* OR nonhuman OR "non human" OR Monkey$ OR rat OR rats OR mouse OR mice OR rabbit OR rabbit OR pig OR pigs OR porcine OR dog OR dogs OR hamster OR hamsters OR fish OR chicken OR chickens OR sheep OR cat OR cats OR raccoon OR raccoons OR rodent* OR horse OR horses OR racehorse OR racehorses OR beagle*))

Ovid MEDLINE(R) and Epub Ahead of Print, In-Process, In-Data-Review & Other Non-Indexed Citations and Daily <1946 to November 28, 2022>

| **#** | **Searches** | **Results** |
| --- | --- | --- |
| 1 | exp Cleft Lip/ or exp Cleft Palate/ | 25448 |
| 2 | ((patient care or primary care or family medicine or family physician* or family doctor* or GP or general pract* or p$ediatr* or nurse or nursing or dental* or Speech or language or hearing or voice or vocal or dental or nutrition* or diet* or surgery or surgical or interdisciplinary or multidisciplinary or care* or manag*) and (cleft palate* or cleft lip$ or CLP or "CL/P" or "cl/cp")).tw,kw,kf. | 13074 |
| 3 | (palatoplast* or palatal repair* or Cleft Palate repair* or cleft lip repair* or cleft care*).tw,kw,kf. | 3064 |
| 4 | or/1-3 | 30044 |
| 5 | *"Patient Reported Outcome Measures"/ or *"patient satisfaction"/ or *"Surveys and Questionnaires"/ or *benchmarking/ or *" treatment outcome"/ or *"Outcome Assessment, Health Care"/ or ("patient-reported outcome measure*" or "PROM$" or PROMIS or survey$ or questionnaire* or instrument or measurement or "treatment outcome*" or "healthcare outcome*" or "health care outcome*").tw,kw,kf. | 4187344 |
| 6 | 4 and 5 | 2964 |
| 7 | *proxy/ or *caregivers/ or ((proxy or proxies or caregiver or carer* or parent$) and reported outcome*).tw,kw,kf. | 33653 |
| 8 | 4 and 7 | 43 |
| 9 | ("CleftQ" or "Cleft-Q").mp. | 30 |
| 10 | 4 and 9 | 23 |
| 11 | *"Quality of Life"/ or (((quality adj2 life) or QoL or craniofacial* or voice* or "oral health") and (measure* or instrument)).tw,kw,kf. | 198760 |
| 12 | 4 and 11 | 850 |
| 13 | 6 or 8 or 10 or 12 | 3489 |
| 14 | limit 13 to (english language and humans) | 2693 |
| 15 | limit 14 to (case reports or comment or duplicate publication or editorial or interview or letter or news or newspaper article) | 204 |
| 16 | 14 not 15 | 2489 |
| 17 | remove duplicates from 16 | 2488 |

Embase Classic+Embase <1947 to 2022 Week 47>

| **#** | **Searches** | **Results** |
| --- | --- | --- |
| 1 | exp Cleft Lip/ or exp Cleft Palate/ | 34882 |
| 2 | ((patient care or primary care or family medicine or family physician* or family doctor* or GP or general pract* or p$ediatr* or nurse or nursing or dental* or Speech or language or hearing or voice or vocal or dental or nutrition* or diet* or surgery or surgical or interdisciplinary or multidisciplinary or care* or manag*) and (cleft palate* or cleft lip$ or CLP or "CL/P" or "cl/cp")).tw,kw,kf. | 17557 |
| 3 | (palatoplast* or palatal repair* or Cleft Palate repair* or cleft lip repair* or cleft care*).tw,kw,kf. | 3974 |
| 4 | or/1-3 | 41098 |
| 5 | *"Patient-Reported Outcome"/ or *"patient satisfaction"/ or *"Questionnaire"/ or *benchmarking/ or *" treatment outcome"/ or *"Outcome Assessment"/ or ("patient-reported outcome measure*" or "PROM$" or PROMIS or survey$ or questionnaire* or instrument or measurement or "treatment outcome*" or "healthcare outcome*" or "health care outcome*").tw,kw,kf. | 5484451 |
| 6 | 4 and 5 | 4397 |
| 7 | *proxy/ or *caregiver/ or ((proxy or proxies or caregiver or carer* or parent$) and reported outcome*).tw,kw,kf. | 31164 |
| 8 | 4 and 7 | 60 |
| 9 | ("CleftQ" or "Cleft-Q").mp. | 64 |
| 10 | 4 and 9 | 51 |
| 11 | *"Quality of Life"/ or (((quality adj2 life) or QoL or craniofacial* or voice* or "oral health") and (measure* or instrument)).tw,kw,kf. | 278820 |
| 12 | 4 and 11 | 1126 |
| 13 | 6 or 8 or 10 or 12 | 5087 |
| 14 | limit 13 to (human and english language) | 3975 |
| 15 | limit 14 to (books or chapter or conference abstract or conference paper or "conference review" or editorial or letter or note) | 920 |
| 16 | 14 not 15 | 3055 |
| 17 | remove duplicates from 16 | 3040 |

APA PsycInfo <1806 to November Week 3 2022>

| **#** | **Searches** | **Results** |
| --- | --- | --- |
| 1 | exp Cleft Palate/ | 548 |
| 2 | ((patient care or primary care or family medicine or family physician* or family doctor* or GP or general pract* or p$ediatr* or nurse or nursing or dental* or Speech or language or hearing or voice or vocal or dental or nutrition* or diet* or surgery or surgical or interdisciplinary or multidisciplinary or care* or manag*) and (cleft palate* or cleft lip$ or CLP or "CL/P" or "cl/cp")).tw,id,mh. | 780 |
| 3 | (palatoplast* or palatal repair* or Cleft Palate repair* or cleft lip repair* or cleft care*).mp. | 24 |
| 4 | 1 or 2 or 3 | 960 |
| 5 | *"Patient-Reported Outcome Measures"/ or *"client satisfaction"/ or *"Questionnaires"/ or *Best Practices/ or *"treatment outcomes"/ or *"health outcomes"/ or ("patient-reported outcome measure*" or "PROM$" or PROMIS or survey$ or questionnaire* or instrument or measurement or "treatment outcome*" or "healthcare outcome*" or "health care outcome*").tw,id,mh. | 1193532 |
| 6 | 4 and 5 | 202 |
| 7 | parents/ or caregivers/ or ((proxy or proxies or caregiver or carer* or parent$) and reported outcome*).tw,id,mh. | 80558 |
| 8 | 4 and 7 | 48 |
| 9 | ("CleftQ" or "Cleft-Q").mp. | 3 |
| 10 | 4 and 9 | 3 |
| 11 | *"Quality of Life"/ or (((quality adj2 life) or QoL or craniofacial* or voice* or "oral health") and (measure* or instrument)).tw,id,mh. | 59326 |
| 12 | 4 and 11 | 59 |
| 13 | 6 or 8 or 10 or 12 | 266 |
| 14 | limit 13 to (human and english language) | 218 |
| 15 | remove duplicates from 14 | 217 |

EBM Reviews - Cochrane Central Register of Controlled Trials – October 2022

| **#** | **Searches** | **Results** |
| --- | --- | --- |
| 1 | exp Cleft Lip/ or exp Cleft Palate/ | 434 |
| 2 | ((patient care or primary care or family medicine or family physician* or family doctor* or GP or general pract* or p$ediatr* or nurse or nursing or dental* or Speech or language or hearing or voice or vocal or dental or nutrition* or diet* or surgery or surgical or interdisciplinary or multidisciplinary or care* or manag*) and (cleft palate* or cleft lip$ or CLP or "CL/P" or "cl/cp")).tw,kw,kf. | 807 |
| 3 | (palatoplast* or palatal repair* or Cleft Palate repair* or cleft lip repair* or cleft care*).tw,kw,kf. | 362 |
| 4 | or/1-3 | 1021 |
| 5 | exp "Patient Reported Outcome Measures"/ or exp "patient satisfaction"/ or exp "Surveys and Questionnaires"/ or exp benchmarking/ or exp " treatment outcome"/ or exp "Outcome Assessment, Health Care"/ or ("patient-reported outcome measure*" or "PROM$" or PROMIS or survey$ or questionnaire* or instrument or measurement or "treatment outcome*" or "healthcare outcome*" or "health care outcome*").tw,kw,kf. | 515201 |
| 6 | 4 and 5 | 355 |
| 7 | exp proxy/ or exp caregivers/ or ((proxy or proxies or caregiver or carer* or parent$) and reported outcome*).tw,kw,kf. | 3220 |
| 8 | 4 and 7 | 2 |
| 9 | ("CleftQ" or "Cleft-Q").mp. | 2 |
| 10 | 4 and 9 | 1 |
| 11 | exp "Quality of Life"/ or (((quality adj2 life) or QoL or craniofacial* or voice* or "oral health") and (measure* or instrument)).tw,kw,kf. | 75790 |
| 12 | 4 and 11 | 57 |
| 13 | 6 or 8 or 10 or 12 | 382 |
| 14 | limit 13 to english language | 368 |
| 15 | remove duplicates from 14 | 359 |

Web of Science Core Collection – November 29, 2022

| 1 | TS=(( "cleft palate*" or "cleft lip$" or palatoplast* or palatal repair* or Cleft Palate repair* or cleft lip repair* or cleft care*)) | 25,924 |
| --- | --- | --- |
| 2 | TS=(("patient-reported outcome measure*" or "PROM$" or PROMIS or survey$ or questionnaire* or instrument or measurement or "treatment outcome*" or "healthcare outcome*" or "health care outcome*")) | 6,099,329 |
| 3 | #2 AND #1 | 2,984 |
| 4 | TS=((proxy or proxies or caregiver or carer* or parent$ AND reported outcome*)) | 259,436 |
| 5 | #4 AND #1 | 373 |
| 6 | TS=(("CleftQ" or "Cleft-Q")) | 43 |
| 7 | #6 AND #1 | 25 |
| 8 | TS=(("Quality of life" or QoL or craniofacial* or voice* or "oral health" AND measure* or instrument)) | 1,283,204 |
| 9 | #8 AND #1 | 4,559 |
| 10 | #9 OR #7 OR #5 OR #3 | 6,602 |
| 11 | TS=((Animal OR animals or Animal Experiment* OR animal model* OR nonhuman OR "non human" OR Monkey$ OR rat OR rats OR mouse OR mice OR rabbit OR rabbit OR pig OR pigs OR porcine OR dog OR dogs OR hamster OR hamsters OR fish OR chicken OR chickens OR sheep OR cat OR cats OR raccoon OR raccoons OR rodent* OR horse OR horses OR racehorse OR racehorses OR beagle*)) | 6,254,772 |
| 12 | #10 NOT #11 | 5,695 |
| 13 | #10 NOT #11 and Article (Document Types) | 5,074 |
| 14 | #10 NOT #11 and Article (Document Types) and English (Languages) | 4,954 |

### Updated Search Results (July 16, 2024)

**Literature Search Strategy**

| **Date** | July 16, 2024 |
| --- | --- |
| **Requester(s)** | ----- , ----- |
| **Topic** | In patients with cleft lip and/or palate, less than 8 years of age, what patient-reported, caregiver proxy-reported, or parent-reported outcome measures have been used to evaluate outcomes of cleft care, and at what age was each type of outcome measure used? |
| **Date limit(s)** | n/a |
| **Language** | English |
| **Other limits** | Human; Omit conference abstracts and other less relevant publication types (embase, cinahl, web of science) |

| **Database [Platform]** *Searches run July 16, 2024* | **Results** |
| --- | --- |
| Ovid MEDLINE(R) and Epub Ahead of Print, In-Process, In-Data-Review & Other Non-Indexed Citations and Daily <1946 to July 16, 2024> | 2783 |
| Embase Classic+Embase [OVID] 1947 to 2024 week 28 | 3525 |
| PsycINFO – 1806 to July week 2 2024 | 243 |
| Cochrane Central Register of Controlled Trials – June 2024 | 443 |
| Web of Science(to be translated) | 5638 |
| **TOTAL* not deduplicated** | **12,632** |

Database(s): **Ovid MEDLINE(R) Epub Ahead of Print and In-Process, In-Data-Review & Other Non-Indexed Citations and Daily** July 16, 2024
Search Strategy:

| **#** | **Searches** | **Results** |
| --- | --- | --- |
| 1 | exp Cleft Lip/ or exp Cleft Palate/ | 26645 |
| 2 | ((patient care or primary care or family medicine or family physician* or family doctor* or GP or general pract* or p$ediatr* or nurse or nursing or dental* or Speech or language or hearing or voice or vocal or dental or nutrition* or diet* or surgery or surgical or interdisciplinary or multidisciplinary or care* or manag*) and (cleft palate* or cleft lip$ or CLP or "CL/P" or "cl/cp")).tw,kw,kf. | 14439 |
| 3 | (palatoplast* or palatal repair* or Cleft Palate repair* or cleft lip repair* or cleft care*).tw,kw,kf. | 3433 |
| 4 | or/1-3 | 31949 |
| 5 | *"Patient Reported Outcome Measures"/ or *"patient satisfaction"/ or *"Surveys and Questionnaires"/ or *benchmarking/ or *" treatment outcome"/ or *"Outcome Assessment, Health Care"/ or ("patient-reported outcome measure*" or "PROM$" or PROMIS or survey$ or questionnaire* or instrument or measurement or "treatment outcome*" or "healthcare outcome*" or "health care outcome*").tw,kw,kf. | 4763460 |
| 6 | 4 and 5 | 3380 |
| 7 | *proxy/ or *caregivers/ or ((proxy or proxies or caregiver or carer* or parent$) and reported outcome*).tw,kw,kf. | 36894 |
| 8 | 4 and 7 | 47 |
| 9 | ("CleftQ" or "Cleft-Q").mp. | 50 |
| 10 | 4 and 9 | 41 |
| 11 | *"Quality of Life"/ or (((quality adj2 life) or QoL or craniofacial* or voice* or "oral health") and (measure* or instrument)).tw,kw,kf. | 223971 |
| 12 | 4 and 11 | 962 |
| 13 | 6 or 8 or 10 or 12 | 3977 |
| 14 | limit 13 to (english language and humans) | 3007 |
| 15 | limit 14 to (case reports or comment or duplicate publication or editorial or interview or letter or news or newspaper article) | 220 |
| 16 | 14 not 15 | 2787 |
| 17 | remove duplicates from 16 | 2783 |

Database(s): **Embase Classic+Embase** 1947 to 2024 Week 28
Search Strategy:

| **#** | **Searches** | **Results** |
| --- | --- | --- |
| 1 | exp Cleft Lip/ or exp Cleft Palate/ | 37140 |
| 2 | ((patient care or primary care or family medicine or family physician* or family doctor* or GP or general pract* or p$ediatr* or nurse or nursing or dental* or Speech or language or hearing or voice or vocal or dental or nutrition* or diet* or surgery or surgical or interdisciplinary or multidisciplinary or care* or manag*) and (cleft palate* or cleft lip$ or CLP or "CL/P" or "cl/cp")).tw,kw,kf. | 19343 |
| 3 | (palatoplast* or palatal repair* or Cleft Palate repair* or cleft lip repair* or cleft care*).tw,kw,kf. | 4436 |
| 4 | or/1-3 | 44050 |
| 5 | *"Patient-Reported Outcome"/ or *"patient satisfaction"/ or *"Questionnaire"/ or *benchmarking/ or *" treatment outcome"/ or *"Outcome Assessment"/ or ("patient-reported outcome measure*" or "PROM$" or PROMIS or survey$ or questionnaire* or instrument or measurement or "treatment outcome*" or "healthcare outcome*" or "health care outcome*").tw,kw,kf. | 6227559 |
| 6 | 4 and 5 | 5014 |
| 7 | *proxy/ or *caregiver/ or ((proxy or proxies or caregiver or carer* or parent$) and reported outcome*).tw,kw,kf. | 37145 |
| 8 | 4 and 7 | 79 |
| 9 | ("CleftQ" or "Cleft-Q").mp. | 93 |
| 10 | 4 and 9 | 81 |
| 11 | *"Quality of Life"/ or (((quality adj2 life) or QoL or craniofacial* or voice* or "oral health") and (measure* or instrument)).tw,kw,kf. | 317462 |
| 12 | 4 and 11 | 1297 |
| 13 | 6 or 8 or 10 or 12 | 5801 |
| 14 | limit 13 to (human and english language) | 4591 |
| 15 | limit 14 to (books or chapter or conference abstract or conference paper or "conference review" or editorial or letter or note) | 1048 |
| 16 | 14 not 15 | 3543 |
| 17 | remove duplicates from 16 | 3525 |

Database(s): **APA PsycInfo** 1806 to July Week 2 2024
Search Strategy:

| **#** | **Searches** | **Results** |
| --- | --- | --- |
| 1 | exp Cleft Palate/ | 595 |
| 2 | ((patient care or primary care or family medicine or family physician* or family doctor* or GP or general pract* or p$ediatr* or nurse or nursing or dental* or Speech or language or hearing or voice or vocal or dental or nutrition* or diet* or surgery or surgical or interdisciplinary or multidisciplinary or care* or manag*) and (cleft palate* or cleft lip$ or CLP or "CL/P" or "cl/cp")).tw,id,mh. | 835 |
| 3 | (palatoplast* or palatal repair* or Cleft Palate repair* or cleft lip repair* or cleft care*).mp. | 25 |
| 4 | 1 or 2 or 3 | 1024 |
| 5 | *"Patient-Reported Outcome Measures"/ or *"client satisfaction"/ or *"Questionnaires"/ or *Best Practices/ or *"treatment outcomes"/ or *"health outcomes"/ or ("patient-reported outcome measure*" or "PROM$" or PROMIS or survey$ or questionnaire* or instrument or measurement or "treatment outcome*" or "healthcare outcome*" or "health care outcome*").tw,id,mh. | 1304833 |
| 6 | 4 and 5 | 230 |
| 7 | parents/ or caregivers/ or ((proxy or proxies or caregiver or carer* or parent$) and reported outcome*).tw,id,mh. | 91073 |
| 8 | 4 and 7 | 50 |
| 9 | ("CleftQ" or "Cleft-Q").mp. | 3 |
| 10 | 4 and 9 | 3 |
| 11 | *"Quality of Life"/ or (((quality adj2 life) or QoL or craniofacial* or voice* or "oral health") and (measure* or instrument)).tw,id,mh. | 65826 |
| 12 | 4 and 11 | 63 |
| 13 | 6 or 8 or 10 or 12 | 297 |
| 14 | limit 13 to (human and english language) | 244 |
| 15 | remove duplicates from 14 | 243 |

Database(s): **EBM Reviews - Cochrane Central Register of Controlled Trials** June 2024
Search Strategy:

| **#** | **Searches** | **Results** |
| --- | --- | --- |
| 1 | exp Cleft Lip/ or exp Cleft Palate/ | 640 |
| 2 | ((patient care or primary care or family medicine or family physician* or family doctor* or GP or general pract* or p$ediatr* or nurse or nursing or dental* or Speech or language or hearing or voice or vocal or dental or nutrition* or diet* or surgery or surgical or interdisciplinary or multidisciplinary or care* or manag*) and (cleft palate* or cleft lip$ or CLP or "CL/P" or "cl/cp")).tw,kw,kf. | 956 |
| 3 | (palatoplast* or palatal repair* or Cleft Palate repair* or cleft lip repair* or cleft care*).tw,kw,kf. | 408 |
| 4 | or/1-3 | 1216 |
| 5 | exp "Patient Reported Outcome Measures"/ or exp "patient satisfaction"/ or exp "Surveys and Questionnaires"/ or exp benchmarking/ or exp " treatment outcome"/ or exp "Outcome Assessment, Health Care"/ or ("patient-reported outcome measure*" or "PROM$" or PROMIS or survey$ or questionnaire* or instrument or measurement or "treatment outcome*" or "healthcare outcome*" or "health care outcome*").tw,kw,kf. | 623563 |
| 6 | 4 and 5 | 468 |
| 7 | exp proxy/ or exp caregivers/ or ((proxy or proxies or caregiver or carer* or parent$) and reported outcome*).tw,kw,kf. | 4620 |
| 8 | 4 and 7 | 3 |
| 9 | ("CleftQ" or "Cleft-Q").mp. | 2 |
| 10 | 4 and 9 | 1 |
| 11 | exp "Quality of Life"/ or (((quality adj2 life) or QoL or craniofacial* or voice* or "oral health") and (measure* or instrument)).tw,kw,kf. | 97273 |
| 12 | 4 and 11 | 72 |
| 13 | 6 or 8 or 10 or 12 | 500 |
| 14 | limit 13 to english language | 483 |
| 15 | remove duplicates from 14 | 443 |

Database(s): Web of Science Core Collection – November 29, 2022

Search Strategy:

| **#** | **Searches** | **Results** |
| --- | --- | --- |
| 1 | TS=(( "cleft palate*" or "cleft lip$" or palatoplast* or palatal repair* or Cleft Palate repair* or cleft lip repair* or cleft care*)) | 28999 |
| 2 | TS=(("patient-reported outcome measure*" or "PROM$" or PROMIS or survey$ or questionnaire* or instrument or measurement or "treatment outcome*" or "healthcare outcome*" or "health care outcome*")) | 6860461 |
| 3 | #2 AND #1 | 3428 |
| 4 | TS=((proxy or proxies or caregiver or carer* or parent$ AND reported outcome*)) | 303107 |
| 5 | #4 AND #1 | 436 |
| 6 | TS=(("CleftQ" or "Cleft-Q")) | 63 |
| 7 | #6 AND #1 | 42 |
| 8 | TS=(("Quality of life" or QoL or craniofacial* or voice* or "oral health" AND measure* or instrument)) | 1458264 |
| 9 | #8 AND #1 | 5121 |
| 10 | #9 OR #7 OR #5 OR #3 | 7491 |
| 11 | TS=((Animal OR animals or Animal Experiment* OR animal model* OR nonhuman OR "non human" OR Monkey$ OR rat OR rats OR mouse OR mice OR rabbit OR rabbit OR pig OR pigs OR porcine OR dog OR dogs OR hamster OR hamsters OR fish OR chicken OR chickens OR sheep OR cat OR cats OR raccoon OR raccoons OR rodent* OR horse OR horses OR racehorse OR racehorses OR beagle*)) | 7061085 |
| 12 | #10 NOT #11 | 6480 |
| 13 | #10 NOT #11 and Article (Document Types) | 5763 |
| 14 | #10 NOT #11 and Article (Document Types) and English (Languages) | 5638 |
