# Supplementary material for: Proxy- and Patient-Reported Outcome Measures in Cleft Lip and/or Palate Under 8 Years of Age: A Scoping Review
Source: Cleft Palate Craniofac J. 2025 Dec 16;63(10):3176–91. doi: 10.1177/10556656251382058 (PMC13542404; doi:10.1177/10556656251382058)
Supplement: Supplementary file 2 [file sj-docx-2-cpc-10.1177_10556656251382058.docx]

### Supplementary Table 2: Data-charting instrument

## General Information

| Author  *Surname of first author* |  |
| --- | --- |
| Year |  |
| Title of Paper |  |
| PMID |  |
| Country of origin | - United States - UK - Canada - Australia - Other   - Write “Other” Text Here |

## Characteristics of Included Studies

### Methods

| Aim of Study |  |
| --- | --- |
| Study Design | - Randomised controlled trial - Non-randomised experimental study - Cohort study - Cross sectional study - Case control study - Systematic review - Qualitative research - Prevalence study - Case series - Case report - Diagnostic test accuracy study - Clinical prediction rule - Economic evaluation - Text and opinion - Instrument or Tool Development/Validation - Other   - Write “Other” Text Here |

Dates

|  | Start Date | End Date |
| --- | --- | --- |
| Month/Year |  |  |

| Inclusion Criteria |  |
| --- | --- |
| Exclusion Criteria |  |

### Overall Participants

#### Numbers

|  | Female | Male | Non-Binary | Total |
| --- | --- | --- | --- | --- |
| Total Sample |  |  |  |  |
| Cleft Subgroup |  |  |  |  |
| <8yrs Cleft subgroup |  |  |  |  |

#### Ages

|  | Age Range | Median/Mean | SD, etc. |
| --- | --- | --- | --- |
| Total Sample |  |  |  |
| Cleft (if subgrp) |  |  |  |

### Cleft Subgroup

#### Cleft Type

Input whatever numbers are present in the paper. We can convert later!

Place 'NR' for those not reported.

|  | Cleft Lip + Palate | Cleft Palate Only | Cleft Lip Only | Cleft Lip w/ Alveolus | Total Participants |
| --- | --- | --- | --- | --- | --- |
| N or % |  |  |  |  |  |

## Outcome Data

|  | Name of Questionnaire | Constructs Measured | Who Reports? | Age-Range |
| --- | --- | --- | --- | --- |
| Questionnaire #1 |  |  |  |  |
| Questionnaire #2 |  |  |  |  |
| Questionnaire #3 |  |  |  |  |
| Questionnaire #4 |  |  |  |  |
| Questionnaire #5 |  |  |  |  |
| Questionnaire #6 |  |  |  |  |
| Questionnaire #7 |  |  |  |  |
| Questionnaire #8 |  |  |  |  |
| Questionnaire #9 |  |  |  |  |
| Questionnaire #10 |  |  |  |  |
